# Molecular characterization of retinal stem cells and their niches in adult zebrafish

**DOI:** 10.1186/1471-213X-6-36

**Published:** 2006-07-26

**Authors:** Pamela A Raymond, Linda K Barthel, Rebecca L Bernardos, John J Perkowski

**Affiliations:** 1 Department of Molecular, Cellular & Developmental Biology, University of Michigan, Ann Arbor, MI 48109, USA; 2 Neuroscience Program, University of Michigan, Ann Arbor, MI 48109, USA

## Abstract

**Background:**

The persistence in adult teleost fish of retinal stem cells that exhibit all of the features of true 'adult stem cells' – self-renewal, multipotency, and the capacity to respond to injury by mitotic activation with the ability to regenerate differentiated tissues – has been known for several decades. However, the specialized cellular and molecular characteristics of these adult retinal stem cells and the microenvironmental niches that support their maintenance in the differentiated retina and regulate their activity during growth and regeneration have not yet been elucidated.

**Results:**

Our data show that the zebrafish retina has two kinds of specialized niches that sustain retinal stem cells: 1) a neuroepithelial germinal zone at the interface between neural retina and ciliary epithelium, called the ciliary marginal zone (CMZ), a continuous annulus around the retinal circumference, and 2) the microenvironment around some Müller glia in the differentiated retina. In the uninjured retina, scattered Müller glia (more frequently those in peripheral retina) are associated with clusters of proliferating retinal progenitors that are restricted to the rod photoreceptor lineage, but following injury, the Müller-associated retinal progenitors can function as multipotent retinal stem cells to regenerate other types of retinal neurons. The CMZ has several features in common with the neurogenic niches in the adult mammalian brain, including access to the apical epithelial surface and a close association with blood vessels. Müller glia in the teleost retina have a complex response to local injury that includes some features of reactive gliosis (up-regulation of glial fibrillary acidic protein, GFAP, and re-entry into the cell cycle) together with dedifferentiation and re-acquisition of phenotypic and molecular characteristics of multipotent retinal progenitors in the CMZ (diffuse distribution of N-cadherin, activation of Notch-Delta signaling, and expression of *rx1, vsx2/Chx10*, and *pax6a*) along with characteristics associated with radial glia (expression of brain lipid binding protein, BLBP). We also describe a novel specific marker for Müller glia, *apoE*.

**Conclusion:**

The stem cell niches that support multi-lineage retinal progenitors in the intact, growing and regenerating teleost retina have properties characteristic of neuroepithelia and neurogenic radial glia. The regenerative capacity of the adult zebrafish retina with its ability to replace lost retinal neurons provides an opportunity to discover the molecular regulators that lead to functional repair of damaged neural tissue.

## Background

The identification and characterization of neural progenitors that produce neurons and glia in the central nervous system is a subject of intense investigation. It is now widely recognized that neural stem cells persist in specialized 'niches' in the adult mammalian forebrain where they generate large numbers of selected types of neurons [1-3]. One of the most intriguing recent discoveries is that these adult neural stem cells exhibit some properties of glial cells [4,5] and that neurons in certain regions of the developing embryonic mammalian and avian brains also derive from radial glia [6]. In the adult brain, the microenvironmental compartments called 'niches' provide an embryonic-like milieu to support the maintenance of neural stem cells with the essential properties of self-renewal and pluripotency, *i.e*. capacity for multi-lineage differentiation [5,7]. Although still poorly understood, some defining characteristics of adult stem cell niches in the brain and elsewhere are beginning to emerge [8-13]. Some common features of neural stem cells and their niches include: prominent cadherin-mediated adhesive junctions, a rich extracellular matrix and contact with a specialized basal lamina via integrin-mediated junctions, close association with blood vessels, cell-surface carbohydrate markers (*e.g*., stage-specific embryonic antigen-1, SSEA-1, also called Lewis X, LeX or leukocyte cluster of differentiation 15, CD15), expression of BLBP (brain lipid binding protein, encoded by the gene *brain-type fatty-acid binding protein 7*, *FABP7*), expression of selected classes of intermediate filament proteins (*e.g*., nestin), responsiveness to extrinsic signals such as IGF (insulin-like growth factor), TGFβ/BMP (transforming growth factorβ/bone morphogenetic protein) family, Wnts, Shh (sonic hedgehog), Notch, and LIF (leukemia inhibitory factor). Probably not coincidentally, these extrinsic regulators represent all of the major families of signaling pathways that are essential for early embryonic development [14].

The neural retina is an embryonic derivative of the forebrain, but unlike the cerebral cortex, adult neural stem cells have not been described in mammalian retina *in vivo*. With the exception of fish and larval amphibians, retinal neurogenesis in vertebrates is completed during embryonic or early postembryonic development [15-17]. Neural progenitors with the capacity to generate retinal neurons can be recognized from the earliest stages of neural induction by regionalized expression of a series of transcriptional regulators that specify retinal identity [18,19]. These early 'eye field transcription factors' include the paired-class homeobox genes *Pax6 *and *Rx*, a member of the *sine oculis *homeobox family, *Six3*, and a LIM-homeobox gene, *Lhx*. Additional members of the paired-class homeobox family, *Chx10 *(known as *vsx2 *in goldfish and zebrafish [20]) and *Crx*, appear at later stages in the developing optic cup [17]. Continued co-expression of these homeobox transcription factors is a distinguishing feature of multipotent retinal progenitor cells [16,21-24].

In larval amphibians and teleost fish, retinal neurogenesis continues postembryonically at the interface between neural retina and ciliary epithelium, a region called the ciliary marginal zone (CMZ) or circumferential germinal zone [25-29]. In these species the CMZ generates the majority of retinal tissue found in the adult eye [26,30], but the specialized cellular and molecular characteristics of the CMZ niche that support the maintenance and regulate the activity of retinal stem cells in the adult fish eye have not yet been elucidated. A reduced and transient CMZ has been described recently in postnatal chicks and marsupial mammals [31] and a residual CMZ is seen in mice with a genetic lesion that increases activity of sonic hedgehog signaling [22]. Although the ciliary epithelium of the adult mammalian retina may retain some neurogenic potential *in vivo *[32] and *in vitro *[33,34], any endogenous capacity for neurogenesis in these species remains latent and without functional consequence.

In addition to the CMZ, another source of retinal progenitor cells persists in the adult teleost fish retina, although the identity and characteristics of these cells are not well understood. In the uninjured, differentiated retina, rod photoreceptors accumulate in central retina as the fish eye grows, and they are generated by rapidly dividing, lineage-restricted rod precursor cells located in the outer nuclear layer (ONL), among the nuclei of differentiated rods [35]. The rod precursors are derived from more slowly proliferating progenitors in the inner nuclear layer (INL) that give rise to clusters of rapidly proliferating progenitors (transit amplifying cells), which migrate into the outer nuclear layer along the radial fibers of the Müller glial cells in larval [35] and adult [36,37] fish. Not all of the Müller glia are associated with neurogenesis and the spatial distribution of rod precursors and clusters of INL progenitors varies in that they are present in greater density near the CMZ, *i.e*., in the most recently generated retina [36,37]. The slowly dividing progenitors in the INL are immunoreactive for Pax6 [37], but it is not known whether they express other retinal progenitor transcription factors found in the CMZ. The rod precursors in the ONL do not express Pax6, but they do express the proneural bHLH transcription factor, *NeuroD*, suggesting that these ONL progenitors may be committed to the photoreceptor lineage [38].

Although their identity has not yet been established, it is clear that multipotent retinal stem cells (with the capacity to generate all types of retinal neurons, not just rod photoreceptors) persist not only in the germinal zone of the CMZ but also in central retina. Adult teleost fish have a robust capacity to regenerate retinal neurons and to restore the appropriate laminar retinal architecture following damage inflicted by surgical lesions, neurotoxins, laser or photic lesions [29,39,40]. All of these studies concluded that the source of regenerated neurons in central retina is an intrinsic progenitor population and not the CMZ. In the intact retina, the progeny of the INL progenitors differentiate only as rod photoreceptors, but several authors have speculated that these progenitors may retain the capacity to generate multiple lineages [29]. Alternatively, it has been suggested that Müller glial cells, which proliferate rapidly in response to retinal damage, may retain a latent capacity to generate neurons in the fish retina [40-42].

In the present study we define the molecular profiles of retinal progenitor cells in the growing and regenerating adult zebrafish retina and we examine the properties of the cellular niches in which they reside. We introduce a simple heat lesion paradigm for analysis of retinal regeneration, we describe the time course of regeneration of cone photoreceptors, and we examine the molecular profile of the injury-induced proliferating progenitor cells that give rise to regenerated cones. Our data show that the adult fish retina has two types of specialized microenvironmental niches that sustain multipotent, self-renewing retinal stem cells: the peripheral CMZ and the microenvironment created by some Müller glia in differentiated retina. We further demonstrate that the retinal stem cell niches in the CMZ germinal zone and the injury-induced stem cell niche in the regenerating retina share a common molecular signature that includes activated Notch signaling, diffuse distribution of N-cadherin, expression of BLBP, and expression of the retinal homeobox genes *rx1, pax6a *and *vsx2/Chx10*. Müller glia form the retinal stem cell niche and may generate retinal stem cells in the regenerating retina. Like the neuroepithelial cells (retinal stem cells and progenitors) of the CMZ they are associated with the specialized basal lamina of blood vessels at the vitreal surface of the retina and their nuclei divide at the apical surface in contact with the subretinal space (an embryonic derivative of the ventricular lumen of the forebrain).

## Results

### Molecular profile of retinal stem cells and progenitor cells in the zebrafish CMZ

**Figure 1:**
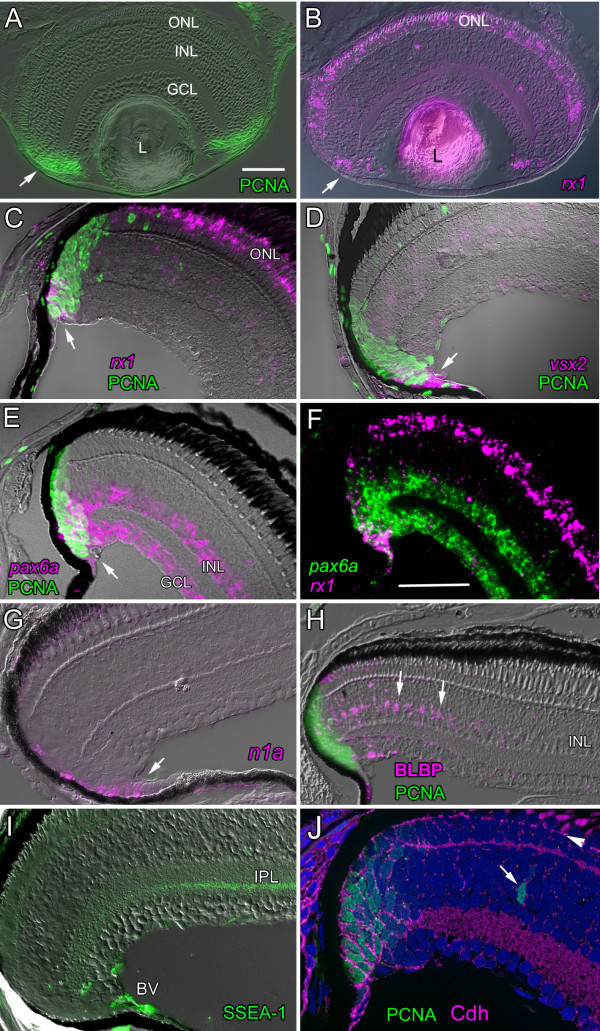
**Molecular characterization of postembryonic neurogenesis in the CMZ of the zebrafish retina**. A) and B) Larval zebrafish (4 dpf). A) Mitotic retinal progenitor cells in the CMZ (arrow) immunoreactive for PCNA (green). B) *rx1 *(magenta) in the CMZ (arrow) and in differentiated cones in the outer nuclear layer (ONL). L, lens; GCL, ganglion cell layer, INL, inner nuclear layer. Scale bar = 50 μm. C) – J) Two-month-old juvenile zebrafish retinas. C) Cells in peripheral CMZ are double-labeled (white arrow) with *rx1 *(magenta) and PCNA (green). D) Cells in peripheral CMZ are double-labeled (white arrow) with *vsx2 *(magenta) and PCNA (green). E) Amacrine cells in the INL and ganglion cells in the GCL express *pax6a*, and many PCNA^+ ^cells in the CMZ are double-labeled with *pax6a *(white arrow). F) Cells in the peripheral CMZ co-express *rx1 *(magenta) and *pax6a *(green). Scale bar = 50 μm. G) Cells in the CMZ (arrow) express *notch1a *(magenta). H) PCNA^+ ^cells (green) in the CMZ, nonpigmented cells in the adjacent ciliary margin and Müller glia (arrows) in the differentiated retina adjacent to the CMZ are BLBP^+^. BLBP-immunoreactivity gradually disappears from Müller glia more centrally. I) Immunoreactivity for SSEA-1/LeX (green) around the circumferential blood vessel (BV) and in a narrow band of the inner plexiform layer, IPL. J) Immunoreactivity for N-cadherin protein (Cdh, magenta) completely surrounds the cell bodies of PNCA^+ ^(green) mitotic cells in the CMZ and cells in the adjacent non-pigmented ciliary epithelium. In the laminated retina, N-cadherin is localized to the zonula adherens junctions of the outer limiting membrane (arrowhead) and synaptic layers. An isolated PCNA^+ ^cell in the inner nuclear layer (arrow) is an INL progenitor in the rod lineage. Nuclei are counterstained with DAPI (blue).

The initial differentiation of retinal neurons and formation of retinal laminae in zebrafish is completed by the end of embryonic development at 3 days post-fertilization (dpf), after which proliferating retinal progenitors are concentrated in a germinal zone at the peripheral margins of the retina in a wedge of cells formed by the converging retinal laminae, called the ciliary marginal zone, CMZ (Fig. 1A). A subset of the proliferating retinal progenitors in the zebrafish CMZ express paired-class homeobox transcription factors associated with retinal specification, including *rx1*, which is also expressed in differentiating (postmitotic) cone photoreceptors in the ONL (Fig. 1B, C, F), *vsx2/Chx10 *(Fig. 1D) and *pax6a *(Fig. 1E). In the most peripheral region of the germinal zone adjacent to the ciliary epithelium, *pax6a *is co-expressed with *rx1 *(Fig. 1F), which is indicative of a multi-lineage capacity.

**Figure 2:**
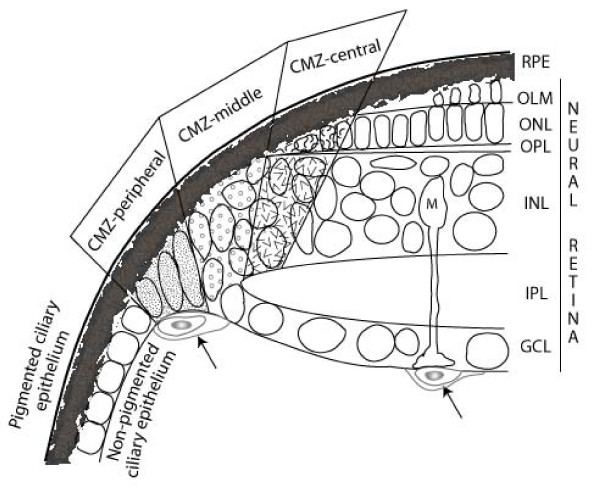
**Definition of CMZ regions for the analysis of regionalized gene expression**. Schematic drawing of histological landmarks that define four areas in the CMZ: peripheral, middle, central-inner and central-outer (shaded regions). The outer plexiform layer (OPL) divides central-inner from central-outer. The definitive ganglion cell layer (GCL) derives from the middle CMZ, reflecting their early birth order. Progenitors in central-inner CMZ are destined for the inner nuclear layer (INL) and the central-outer region produces cone photoreceptors in the outer nuclear layer (ONL). Arrows, blood vessels; RPE, retinal pigmented epithelium; OLM, outer limiting membrane; IPL, inner plexiform layer; M, Müller glial cell.

Figure 1A–E shows that the territory occupied by PCNA^+ ^proliferating cells in the CMZ is wider than the expression domains of the retinal progenitor transcription factors. In larval Xenopus retina the pattern and spatial distribution of these and other molecular markers in progenitor cells in the CMZ has been interpreted to reflect the temporal sequence of retinal development: multipotent progenitors (*i.e*. retinal stem cells) are adjacent to the ciliary epithelium and retinal progenitors with more restricted fates are more centrally located [43]. To determine whether a similar organization exists in the CMZ in zebrafish we systematically examined the expression patterns of a selected subset of these genes. We divided the CMZ into four anatomically defined regions: peripheral, middle, central-outer and central-inner (Fig. 2). We then assayed gene expression by *in situ *hybridization in the CMZ of 2-month-old zebrafish and ranked the level of signal in each of the four regions as strong (rank = 3), moderate (2), weak (1), or not detectable (0). Because *in situ *hybridization is not a quantitative assay tool, no conclusions can be drawn about absolute levels of gene expression, nor can the absence of a signal be taken as evidence of no expression, since other more sensitive techniques detect lower levels of mRNA transcripts. However, the advantage of *in situ *hybridization is that it provides spatial information about relative expression levels in different cell populations within a heterogeneous tissue, which was the primary goal of our analysis. The results are presented in Table 1.

**Table 1:** Relative strength of in situ hybridization signals in the CMZ. The level of signal was estimated as strong (rank = 3), moderate (2), weak (1), or not detectable (0) in the four regions of CMZ (defined in Fig. 2; Peri. = Peripheral, Mid. = Middle, Cent. = Central). For each probe, 11 to 32 sections from 2 to 4 retinas were evaluated. The arithmetic means of the ranks are reported and the data for each probe are ordered by descending rank in the peripheral CMZ. Values greater than 1.0 are bolded for emphasis.

**Probe**	**Peri**.	**Mid**.	**Cent. Inner**	**Cent. Outer**
***pax6a***	**2.2**	**2.3**	**2.4**	0
***rx1***	**2.0**	0	0.2	**2.0**
***vsx2***	**1.9**	0.2	0	0
***notch1a***	**1.9**	**1.4**	0	0
***her6***	**1.9**	0.2	0.1	0
***notch1b***	**1.7**	**1.4**	0	0
***deltaC***	**1.7**	0.3	0	0.6
***ascl1a***	**1.7**	0.6	0	0
***rx2***	**1.7**	0	0	**1.7**
***her2***	**1.4**	0.2	0	0.1
***neuroD***	0.3	0.3	0.8	**2.0**

In the peripheral region of the germinal zone *rx1, vsx2/Chx10*, and *pax6a *show the highest signal levels of all the probes we examined (Table 1). Expression of *pax6a *is moderately high in the peripheral germinal zone and remains strong in middle and central-inner regions, consistent with the continued expression of this gene in differentiated amacrine cells. The level of *rx1 *signal is moderate in the peripheral germinal zone, and *rx2 *is slightly weaker. Both *rx *genes are sharply down-regulated in the middle germinal zone, but reappear in the central-outer region in differentiating cone photoreceptors. The profile of *vsx2/Chx10 *is similar to *rx1/rx2 *in that expression levels are highest in the peripheral CMZ and are sharply down-regulated in the middle CMZ. Expression of *vsx2/Chx10 *reappears in the differentiated INL, presumably in bipolar cells, but outside the CMZ [20]. The expression domain of these transcription factors extends from the CMZ into the adjacent ciliary epithelium (Fig. 1C–F).

Other transcriptional regulators associated with retinal progenitors in several vertebrate species are members of the basic helix-loop-helix (bHLH) family of proneural genes [21]. A zebrafish ortholog of mammalian/chick *Mash1/Cash1*, called *ascl1a *(formerly *zash1a*) has a moderate signal level in the peripheral CMZ, is reduced in the middle and is absent from central regions (Table 1). Proneural genes related to the *atonal *family, such as *neuroD*, are typically activated at later stages in the neuronal differentiation cascade and are associated with determination of specific neuronal cell types [44]. In the retina, *neuroD *specifies photoreceptors in zebrafish [38]. The *neuroD *probe produces only a very weak signal in the peripheral and middle CMZ, but increases sharply in the central regions, especially the outer part, where photoreceptors are differentiating (Table 1). All of these results are consistent with the idea that the most primitive (*i.e*., multipotent) retinal stem cells are located in the most peripheral region of the CMZ adjacent to the ciliary epithelium and more restricted retinal progenitors lie closer to the differentiated retina.

The Notch-Delta signaling pathway plays an important role in cell fate choice in the retina and in particular the decision of whether to differentiate as a neuron or a Müller glia or to remain undifferentiated [45,46]. Several constituents of this bidirectional signaling pathway are expressed in the CMZ in larval and juvenile zebrafish, including the Notch ligands *deltaA, deltaB, deltaC, deltaD*, several Notch family members (*notch1a, notch1b*, *notch 3*) and downstream mediators of activated Notch, *her6*, the zebrafish ortholog of mammalian *Hes1 *and *her2/Hes5 *(Fig. 1G and data not shown). Analysis of relative expression levels shows that in the most peripheral region of the CMZ components of the Notch-Delta signaling cascade give moderate signals (Table 1). Expression levels are lower in the middle and central regions of the CMZ. The signals for *notch3 *are similar to the *notch1 *paralogs (data not shown). These data indicate that the Notch-Delta signaling pathway is activated in retinal progenitors including multipotent retinal stem cells that co-express a set of transcriptional regulators implicated in maintenance of multipotency and specification of retinal cell fate.

### The microenvironment of the zebrafish CMZ has features typical of neural stem cell niches

The radial glia/astrocyte marker BLBP has been associated with adult neural stem cell niches in mammalian brain [47]. We used a polyclonal antibody against a synthetic peptide of human BLBP that is 90% identical to the predicted amino acid sequence of the orthologous zebrafish gene, *fabp7a *[48]. We show that retinal stem cells and progenitors in the CMZ and immature Müller glial cells are immunoreactive for BLBP (Fig. 1H). BLBP immunoreactivity extends throughout the CMZ and into the adjacent ciliary epithelium. In larval zebrafish retina (1-week-old), the CMZ and immature Müller glia distributed across the retina are BLBP^+ ^(data not shown), but in 2-month-old juvenile fish only the recently generated, immature Müller glial cells closest to the CMZ are labeled (Fig. 1H). Thus, the BLBP protein is down-regulated with maturation of zebrafish Müller glia.

In mammals, the extracellular matrix-associated carbohydrate epitope SSEA-1/LeX/CD15 is a surface marker for embryonic stem cells and adult neural stem cells [12] as well as subpopulations of embryonic retinal progenitor cells [49]. The antigen is the trisaccharide, 3-fucosyl-N-acetyl-lactosamine, which is a component of membrane glycolipids and glycoproteins from a variety of tissues. In zebrafish, the SSEA-1 epitope is present at high levels around the circumferential blood vessel [28,50] that lies on the vitreal surface at the border between neural retina and ciliary epithelium overlying the CMZ (Fig. 1I) and around other blood vessels that line the vitreal surface (data not shown). A second site of high expression is a discrete laminar zone of undetermined identity in the inner plexiform layer (Fig. 1I), potentially on amacrine cell processes [51]. The retinal pigmented epithelium (RPE) also expresses SSEA-1 (data not shown).

The calcium-dependent, homophilic cell adhesion protein N-cadherin (encoded by the gene *Cadherin-2*) is diffusely distributed on the basolateral plasma membranes of retinal stem cells and progenitors in the CMZ and adjacent ciliary epithelium (Fig. 1J), similar to the distribution of N-cadherin on undifferentiated neuroepithelial progenitors in the embryonic retinal primordium [52]. Proliferating retinal progenitor cells in the CMZ are ensheathed in a network of cadherin-mediated adhesive interactions with neighboring cells. In contrast, in the differentiated retina, N-cadherin is targeted to the zonula adherens that form the outer limiting membrane (apical surface of the retina), to the synaptic (plexiform) layers and to axons of retinal ganglion cells (Fig. 1J and [52]).

### Progenitors with the molecular profile of retinal stem cells are activated locally in differentiated retina by thermal lesions

**Figure 3:**
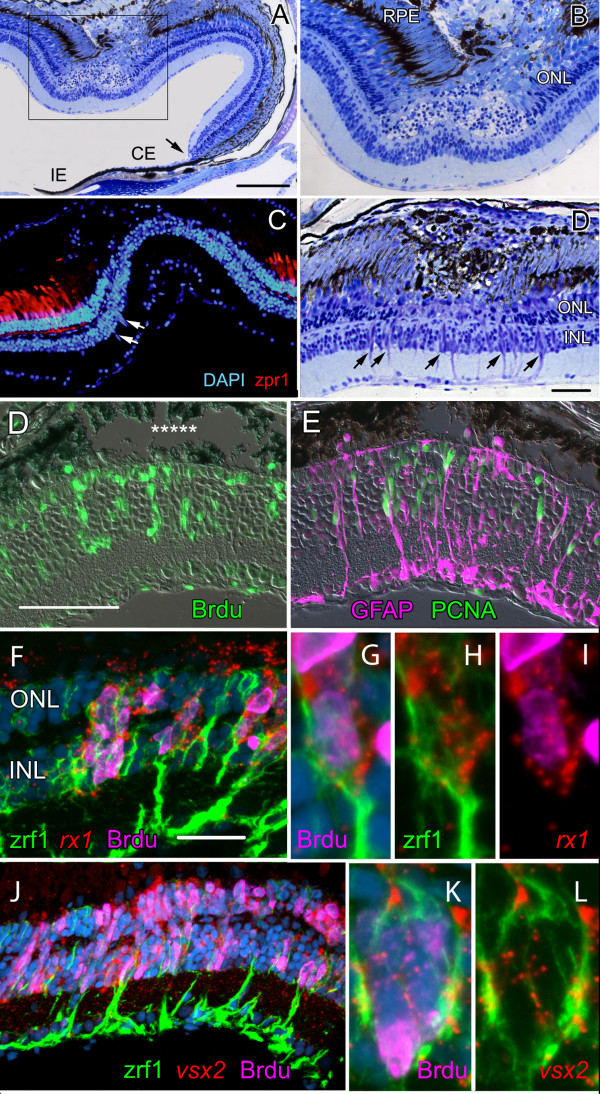
**Injury-induced proliferating retinal progenitors express *rx1, vsx2*, and *pax6a***. A) – D) Histology of the heat-lesioned retina at 1 and 3 dpl; the boxed area in A) is magnified in B). Cell loss is mainly confined to the retinal pigmented epithelium (RPE) and photoreceptors in the outer nuclear layer, ONL. The CMZ (arrow in A) is at the junction between the neural retina and the ciliary epithelium (CE), which is continuous anteriorly with the iris epithelium (IE). Scale bar (A) = 150 μm. C) At 3 dpl double cones immunoreactive with zpr1 (red) are missing from the lesioned area and elongated nuclei appear in the inner nuclear layer (white arrows). Counterstained with DAPI (blue). D) By 3 dpl, radial fibers of Müller glia in the inner nuclear layer, INL, are visible in the region of the lesion (black arrows), indicative of reactive gliosis. Scale bar = 50 μm. D) Proliferating cells in the inner and outer nuclear layers of the retina in the lesioned area (asterisks) at 5 dpl incorporated BrdU (green) injected at 4 dpl. E) Injury-induced proliferating cells are also immunoreactive for proliferating cell nuclear antigen, PCNA (green) and are associated with radial fibers of Müller glia (magenta, anti-human GFAP). Note: the commercial polyclonal GFAP antibody used here is not selective for GFAP in zebrafish but labels other intermediate filaments (data not shown). In contrast, the monoclonal zrf1, which was generated against zebrafish proteins, selectively labels zebrafish GFAP [97]. Scale bar = 50 μm. F) – L) A 4 h or 24 h pulse of BrdU at 4 or 5 dpl labels clusters of nuclei (magenta) that extend between the INL and ONL and express *rx1 *(F) and *vsx2 *(J) as visualized by *in situ *hybridization (red). DAPI (blue); zrf1 (green, anti-zebrafish GFAP). Scale bar = 25 μm. Higher magnification views of *rx1 *(G,H, I) and *vsx2 *(K, L) in BrdU^+ ^progenitors enclosed in a basket of zrf1^+ ^Müller glial fibers.

To determine whether retinal stem cells responsible for retinal regeneration following injury have molecular profiles similar to those in the CMZ, we created local thermal lesions largely limited to the RPE and underlying photoreceptors in the retina of adult zebrafish (Fig. 3A–D). The diameter of the lesioned area ranges from ~200 to 400 μm. Loss of photoreceptors is apparent within 1 day post-lesion (dpl) and pyknotic nuclei fill the ONL and subretinal space between neural retina and RPE (Fig. 3A, B). Destruction of cone photoreceptors within the lesion is revealed by the absence of immunoreactivity with zpr1 (Fig. 3C), a highly specific cell-surface marker for zebrafish double cones (pairs of red and green cones joined by specialized cell-cell junctions [53]).

Thermal lesions trigger a local increase in mitotic activity in the retina, which begins within 2 dpl (data not shown). In lesioned areas elongated clusters of cells associated with radial fibers of Müller glia appear by 3 dpl (Fig. 3D, E). Cells in these clusters incorporate BrdU (Fig. 3D). We confirmed that incorporation of BrdU reflects mitotic cell division and not DNA repair in response to damage [54] by immunostaining for proliferating cell nuclear antigen, PCNA (Fig. 3E). These mitotic cells are closely associated with radial fibers of Müller glia, all of which are strongly immunoreactive with antibodies against glial fibrillary acidic protein, GFAP (Fig. 3E). Nuclei of the activated Müller glia also reenter the cell cycle and migrate from the INL into the ONL (RLB and PAR, unpublished observations), as we showed previously in regenerating goldfish retina [42,55].

At 4 and 5 dpl the injury-induced proliferating cells associated with GFAP^+ ^radial Müller fibers co-express at high levels the retinal stem cell markers *rx1, vsx2/Chx10 *(Fig. 3F–L) and *pax6a *(data not shown). BrdU^+ ^mitotic clusters show strong *rx1 *(Fig. 3F–I) and *vsx2/Chx10 *signals (Figs. 3J–L). The expression of these transcription factors within the lesion extends vertically beyond the laminar boundaries of their respective expression domains in the differentiated retina (Fig. 1C–F).

**Figure 4:**
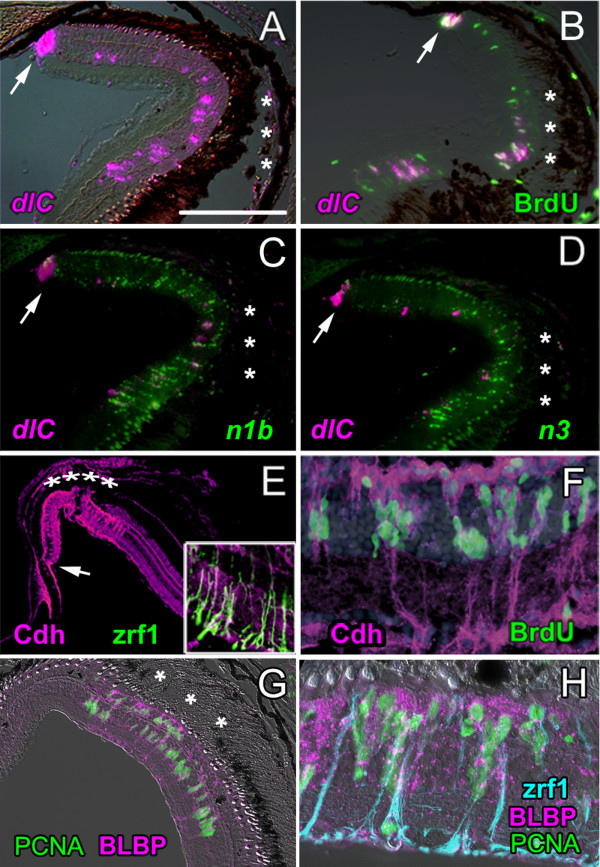
**Molecular profile of the injury-induced proliferating retinal progenitors is similar to retinal stem cells in the CMZ**. A) At 4 dpl *deltaC *expression (*dlc*; magenta) is upregulated in the CMZ (arrow) and in cells in the INL beneath the lesion (asterisks). Scale bar = 150 μm. B) At 4 dpl, cells in the CMZ (arrow) and lesioned area labeled with a 2-hour pulse of BrdU (green) also express *deltaC *(magenta). C) *notch1b *(*n1b*; green) and D) *notch3 *(*n3*; green) are also up-regulated but are generally not co-expressed with *deltaC *(magenta). E) At 4 dpl, N-cadherin immunoreactivity (Cdh2; magenta) is strongly up-regulated in the lesioned area (asterisks) and diffusely localized. Inset: zrf1, anti-zebrafish-GFAP (green) in radial fibers of Müller glia in the lesioned retina (7 dpl) co-localizes with N-cadherin immunoreactivity (magenta). F) At 7 dpl, BrdU^+ ^nuclei (green) associate with Müller glial radial fibers that are strongly immunoreactive for N-cadherin (magenta). DAPI (blue). G) At 3 dpl, activated Müller glia confined to the lesioned region (asterisks) express BLBP (magenta), a marker of immature Müller glia, and they are mitotically active (PCNA^+^, green). H) Radial fibers of injury-activated Müller glia are zrf1^+ ^(blue). Proliferating Müller nuclei are PCNA^+ ^(green) and many have migrated to the apical surface (the former ONL where photoreceptors are missing) and are BLBP^+ ^(magenta).

Constituents of the Notch-Delta signaling pathway are also strongly upregulated in the injured retina after thermal lesions (Fig. 4A–D). Between 4 and 7 dpl, *deltaC, notch1b *and *notch3 *probes produce signals in the lesion area comparable in strength to those in the CMZ. Some (but not all) BrdU^+ ^cells in the lesion area at 4 dpl co-label with *deltaC *(Fig. 4B). The *deltaC *and *notch *signals are largely in adjacent cells and are not co-localized (Fig. 4C, D). The *deltaA, deltaB, deltaD*, and *her6/Hes1 *probes similarly produce strong signals in the lesion area (data not shown). In summary, injury-induced proliferating cells closely associated with radial fibers of Müller glia express retinal stem cell transcription factors and activate Notch signaling.

### Injury induces a retinal stem cell niche that recreates the microenvironment of the CMZ

Müller glial cells activated by the lesion acquire several features characteristic of retinal stem/progenitor cells in the CMZ and in the embryonic retina. Activated Müller glial cells up-regulate expression of N-cadherin and the protein becomes distributed diffusely throughout the plasma membrane as it is in retinal stem cells and progenitors in the CMZ (Fig. 4E). The inset in Fig. 4E shows radial fibers of activated Müller glia co-labeled with a monoclonal antibody specific for zebrafish GFAP (zrf1) and polyclonal antibodies against zebrafish N-cadherin. In contrast, in the uninjured differentiated zebrafish retina, N-cadherin immunoreactivity primarily localizes to the adherens junctions formed by Müller glia at the apical surface of the retina (the outer limiting membrane), to the synaptic neuropil of the inner and outer plexiform layers, and to retinal ganglion cell axons [56]. The injury-induced clusters of BrdU^+ ^nuclei are tightly associated with radial Müller fibers that are immunoreactive for N-cadherin (Fig. 4F).

Activated Müller glia in the region of the lesion also up-regulate expression of BLBP (Fig. 4G, H), which is characteristic of retinal stem/progenitor cells in the CMZ and immature Müller glia (Fig. 1H). The boundary between BLBP^+ ^and BLBP^- ^Müller glia is very abrupt (Fig. 1G), which suggests that only the Müller glia directly impacted by the retinal injury dedifferentiate and reacquire an immature molecular profile.

### Cone photoreceptors regenerate within a week

Some of the mitotically active cells induced by the thermal lesion are retinal stem/progenitor cells that differentiate into retinal neurons, thus repairing the retinal damage. To identify the retinal stem cells responsible for regenerating retinal neurons, we first needed to know when the regenerated neurons are born so we could focus on cells proliferating during and/or before that time. Not all of the mitotic cells at every interval following the lesion are retinal stem cells; for example, microglia proliferate rapidly in response to retinal injury [42,57] and phagocytize the dead cells and debris. Differentiated Müller glia proliferate after injury, even in mammalian retinas that fail to regenerate neurons [58].

**Figure 5:**
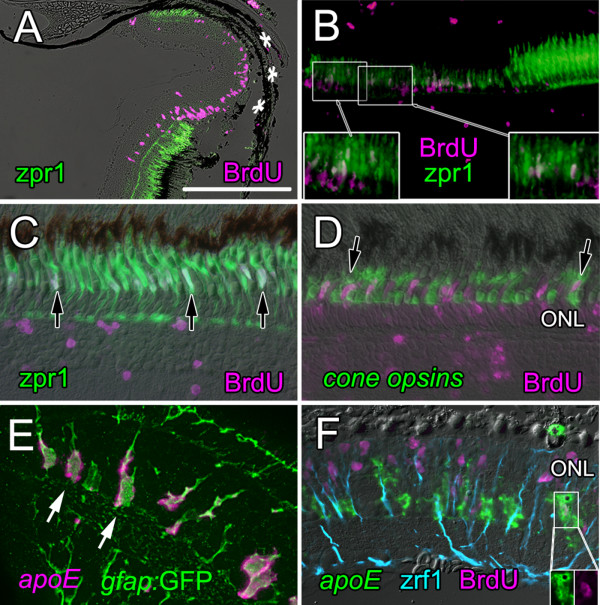
**Injury-induced retinal progenitors regenerate cone photoreceptors within a week**. A) At 4 dpl, proliferating cells (BrdU; magenta) fill the lesioned area (asterisks) in which double cones immunoreactive for zpr1 (green) are missing. Scale bar = 250 μm. B) By 7 dpl, some retinal progenitors that were labeled with BrdU (magenta) at 4 dpl have begun to differentiate into cones and are double-labeled with zpr1 (green). Boxed areas are shown at higher magnification in the insets; double-labeled cells are white. C) By 31 dpl fully differentiated, regenerated cone photoreceptors (zpr1; green) are labeled with BrdU (magenta) injected at 3 dpl (double-labeled white nuclei are indicated by black arrows). Unidentified BrdU^+ ^nuclei are seen in the inner retina. D) Cocktail of riboprobes to cone opsins (green) identifies BrdU^+ ^(magenta), regenerated cones at 31 dpl (double-labeled white nuclei are indicated by black arrows). BrdU^+ ^rod nuclei (magenta) are in the inner part of the outer nuclear layer, ONL. E) Müller glia in a transgenic zebrafish Tg(*gfap*:GFP)^mi2001 ^are labeled with anti-GFP (green) and co-express *apoE *(magenta, *in situ *hybridization) in their cell bodies. F) At 4 dpl, most BrdU^+ ^proliferating nuclei are in the outer nuclear layer, ONL, but a few *apoE*^+ ^Müller glial cells are also BrdU^+ ^(inset). Radial fibers of Müller glia are labeled with zpr1/anti-GFAP (blue).

In this study, we confined our analysis of the time course of neuronal regeneration in the retina to cone photoreceptors because they have distinctive and highly specialized morphology and several unique cell-specific markers. To document regeneration of cones, we first labeled proliferating cells with BrdU at 3 to 10 dpl and then waited for several days up to one month to allow the progeny of the mitotic cells to differentiate. We then identified double-cones with the zpr1 antibody (Fig. 3C). When the retina is examined 4 hours after BrdU exposure at 4 dpl, mitotic cells are labeled in the region of the lesion (Fig. 5A). At this stage, microglia (the vascular-derived, resident macrophages that are specifically labeled with the 4C4 antibody [59]) are in the subretinal space between the outer limiting membrane and the RPE and are not part of the pool of proliferating cells within the retina (data not shown). When fish survived for 4 days after the BrdU (to 7 dpl), a few progeny of the BrdU^+ ^cells had begun to differentiate and express the double cone marker zpr1, and they are small and immature compared with the intact cones adjacent to the lesion (Fig. 5B). By 31 dpl the regenerated cones are fully differentiated and indistinguishable from the surrounding photoreceptors, with the exception that their nuclei retain the BrdU-label (Fig. 5C). In all lesions at long survival intervals, persistent BrdU^+ ^nuclei are seen in the INL (Fig. 5C). We do not know the identity of these cells, although they do not have the morphological characteristics of Müller glia and they are not immunoreactive with the microglia-specific antibody, 4C4 (data not shown). We presume that most of them are retinal neurons, which were regenerated following loss due to the lesion, as has been well documented in several other models of retinal regeneration in teleost fish [29,39].

**Figure 6:**
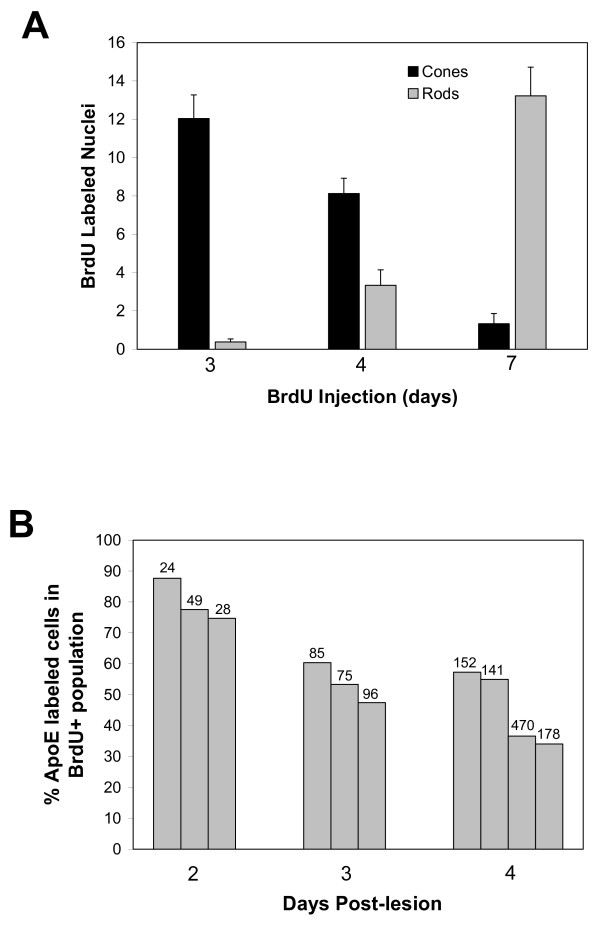
**A) Time course of regeneration of cone and rod photoreceptors**. The number of BrdU^+ ^rod and cone photoreceptors per section at 31 dpl is plotted as a function of time of BrdU injection (in days post-lesion). Each bar is the mean number per section calculated by combining data from counts on two retinas and 4 to 17 sections per retina. The data were pooled after an ANOVA showed that there was no significant difference between the average counts between retinas at each time point. The error bars represent one SEM. Comparison of means with a single factor ANOVA showed that slightly more cones are born at 3 dpl than at 4 dpl (p < 0.05), fewer at 7 dpl (p < 0.001) and almost none at 10 dpl (data not shown). In contrast, significantly fewer rods than cones are born at 3 and 4 dpl (p < 0.001), but many more rods than cones are born at 7 (p < 0.001) and 10 dpl (data not shown). **B) Most of the proliferating cells at 2 dpl are Müller glia**. Fish with heat lesioned retinas were exposed to a 4 h pulse of BrdU at 2, 3 or 4 dpl. Retinas were processed for *in situ *hybridization with an *apoE *probe to label cell bodies of Müller glia and for BrdU immunocytochemistry. Each bar represents one lesion, and 2 to 9 sections were counted for each lesion. All BrdU^+ ^nuclei were counted and scored for *apoE*. The mean number of BrdU^+ ^nuclei per section is given above each bar. Statistical analysis of the data by single factor ANOVA indicates that the percent *apoE*^+ ^cells in the BrdU^+ ^population at 2 dpl greater than at 3 dpl (p < 0.01) or at 4 dpl (p < 0.01) but 3 dpl does not differ from 4 dpl.

To determine the time course of cone photoreceptor regeneration, we labeled proliferating cells with BrdU at 3, 4, 7 or 10 dpl, and counted double-labeled cone photoreceptors at 31 dpl. Because the zpr1 antibody labels only the red-green double cones and zebrafish have two additional single cone types that express either blue or ultraviolet-sensitive opsin, we used a cocktail of RNA probes to the four cone opsin classes – red (*lws-1*), green (*rh2-1*), blue (*sws2*) and ultraviolet (*sws1*) – to label all cone types (Fig. 5D). We also counted BrdU^+ ^rod photoreceptors in the ONL in the area of the lesion (Fig. 5D). Cell counts (Fig. 6A) show that regenerated cones in zebrafish are born between 3 and 7 dpl whereas most rods are born at 7 dpl or later (Fig. 6A and data not shown). In the developing zebrafish retina, cones are born before rods as they are in the embryonic mammalian retina [60,61]. In our previous studies of retinal regeneration in goldfish we found that the ongoing production of rod photoreceptors from mitotic rod precursors largely ceases while the complement of cones is restored [62,63] and here we show that a similar process occurs in zebrafish. This temporal shift in the distribution of photoreceptor cell types generated by the retinal stem cells suggests that they respond to changing microenvironmental factors similar to those found in the developing embryonic retina, which regulate the sequential production of retinal neurons.

### Identity of the injury-activated retinal stem cells/progenitors

The longitudinal birthdating analysis identified cells proliferating between 3 and 7 dpl as retinal progenitors whose progeny differentiate into cone photoreceptors. Since cone photoreceptors are not normally produced in the differentiated regions of the intact retina, these data demonstrate that multipotent retinal stem cells persist in central retina with the capacity to respond to injury by altering their lineage profile to compensate for loss of specific neuronal subtypes. As described above, most of the mitotic activity in the lesion area at this time is in elongated clusters of proliferating cells in the INL closely associated with the radial fibers of Müller glia (*e.g*., Fig. 3E–L, 4F, H). These proliferating cell clusters have been observed in all previous studies of retinal regeneration in teleost fish, and their similarity to the progenitors in the rod photoreceptor lineage of the uninjured retina has been noted [64]. Another potential source of latent retinal stem cells are the Müller glia themselves [29,41,42,65,66]. This question has been difficult to resolve because of the tight association of retinal progenitors in the rod lineage with the radial fibers of Müller glia in both the uninjured retina [35] and the regenerating retina (Figs. 3E–L, 4F, H), and the complexity of Müller processes that enwrap neighboring cells [67]. These intimate and complex anatomical relationships make it difficult to determine with fluorescent microscopy and the standard, cytoplasmic Müller-specific markers, *e.g*. GFAP, cellular retinaldehyde binding protein (CRALBP) and glutamine synthetase, whether a given BrdU^+ ^nucleus belongs to the Müller cell or to an associated progenitor.

In an attempt to examine this question with better techniques, we sought a Müller-specific marker that is confined to the cell body. Apolipoprotein E (ApoE) is a small protein component of several different lipoproteins that is secreted by many cells including glia [68]. To determine whether *apoE *is expressed by Müller cells, we used transgenic zebrafish [69] in which enhanced green fluorescent protein (GFP) is controlled by transcriptional regulatory elements of the zebrafish *gfap *gene. The GFP reporter in this transgenic line is cytoplasmic and fills the cell body and all the processes of Müller glia. We found that zebrafish *apoE *is expressed selectively and at high levels in *gfap*: GFP^+ ^differentiated Müller glia, and the signal is largely confined to the cell bodies in the INL (Fig. 5E). The *apoE *probe also co-localizes with a probe that hybridizes to *rlbp1-l *(*retinaldehyde binding protein 1-like*) the zebrafish orthologue of the Müller glial marker CRALBP (cellular retinaldehyde binding protein) [70] (data not shown). It is therefore a useful marker to identify BrdU^+ ^proliferating Müller glia.

If Müller glia function as retinal progenitors/stem cells in the injured retina, they should be proliferating before and/or during the interval when regenerated cone photoreceptors are being born (*i.e*., undergoing their terminal mitotic division), which we found is between 2 and 4 dpl. Very few cells incorporate BrdU at 1 dpl (data not shown), but at 2 dpl approximately 80% of the proliferating cells are *apoE*^+^/BrdU^+ ^Müller glia (Fig. 6B). The fraction of *apoE*^+^/BrdU^+ ^Müller glia in the population of proliferating cells dropped to ~50% by 3 and 4 dpl, while the total number of BrdU^+ ^nuclei increased from a mean of 34 per section at 2 dpl, to 85 per section at 3 dpl, to 235 per section at 4 dpl (Fig. 6B). These data are consistent with several interpretations. Müller glia that express *apoE *constitute the vast majority (80%) of the proliferating cells at 2 dpl when the size of the BrdU^+ ^population is smallest. The remaining 20% of the BrdU^+ ^cells that are *apoE*^- ^at this time could be progenitors in the rod lineage or dedifferentiated Müller glia or progeny of Müller glia that have down-regulated *apoE *expression. A closer examination of the rare BrdU^+ ^cells at early intervals prior to 2 dpl with specific markers for retinal stem cells and progenitors (*e.g., rx1, pax6a, vsx2, neuroD, crx*) might answer this question. The decline in the Müller glia fraction of the expanding BrdU^+ ^population at 3 and 4 dpl could be explained by dedifferentiation of proliferating Müller cells and/or a more rapid rate of proliferation of non-Müller retinal progenitors. Finally, these data are also consistent with the hypothesis that injury-induced retinal stem cells might derive from Müller glia that begin to proliferate at or before 2 dpl, dedifferentiate and give rise to retinal stem cells.

## Discussion

**Figure 7:**
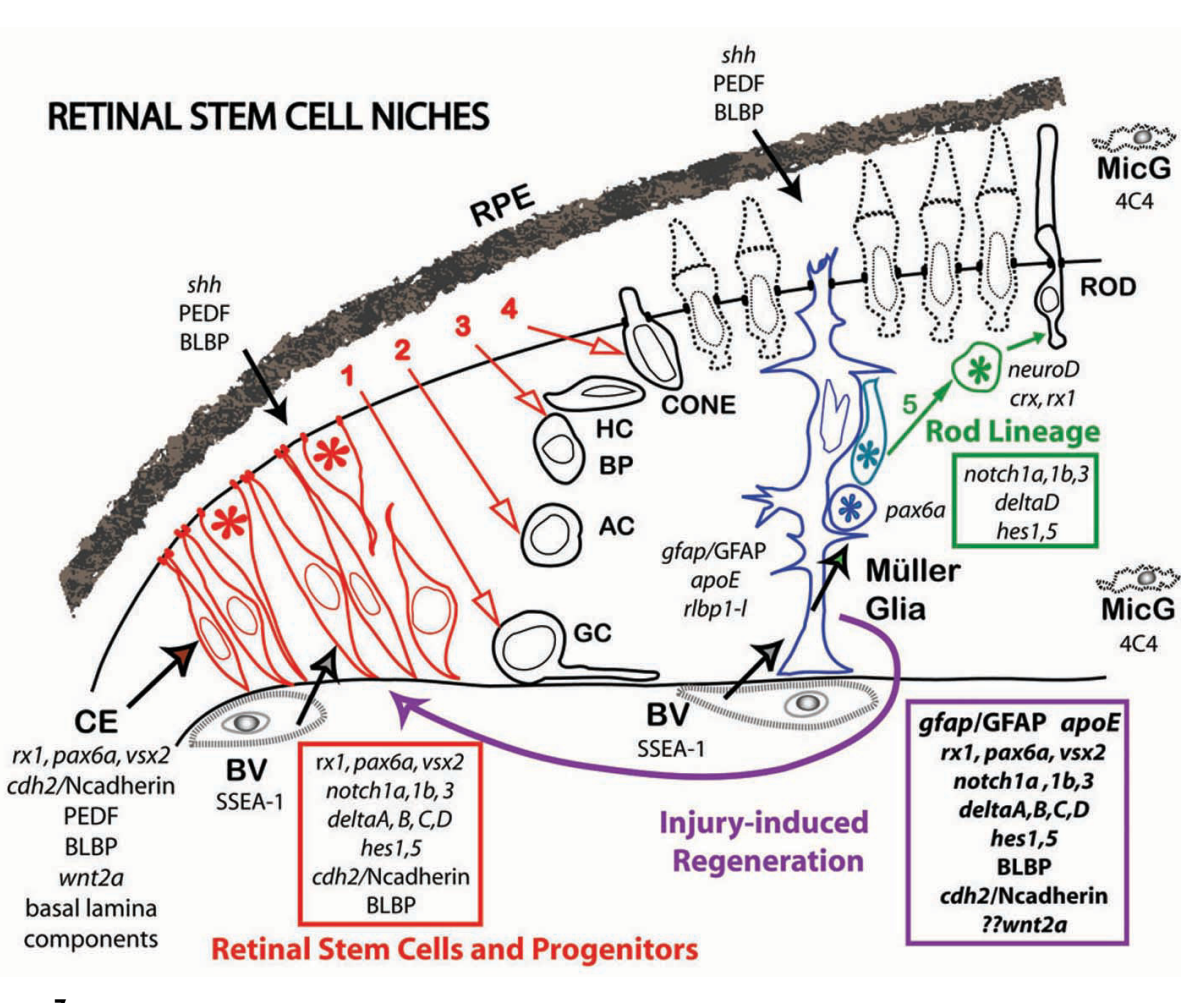
**Stem cell niches in zebrafish retina**. The germinal zone at the boundary between neural retina and ciliary epithelium (CE) is a circumferential wedge of neuroepithelial cells (in red) called the ciliary marginal zone (CMZ). Multipotent retinal stem cells span the width of the retinal epithelium adjacent to the CE and more restricted retina progenitors give rise sequentially to 1) retinal ganglion cells (GC), 2) amacrine cells (AC), 3) bipolar (BP) and horizontal cells (HC), and 4) cone photoreceptors. The CMZ is separated from the retinal pigmented epithelium (RPE) by a narrow subretinal space. The ora collection blood vessel (BV) encircles the retina at the CMZ; other blood vessels of the hyaloid circulation lie along the vitreal surface. Müller glia (blue) span the width of the retina and create a niche that supports retinal progenitors of the last-born retinal neuron in the 5) rod photoreceptor lineage (green). Expression of specific markers is shown for retinal progenitors/stem cells and associated cell types. Retinal injury induces a reorganization of the Müller cell/rod lineage niche to produce a regeneration niche (purple) that mirrors the CMZ niche in patterns of cellular organization and gene expression.

We have characterized the molecular microenvironment of two kinds of specialized niches that sustain retinal stem cells in zebrafish: 1) the ciliary marginal zone (CMZ) at the peripheral retinal margin and 2) some Müller glia in the differentiated retina (Fig. 7). The retinal stem cell niche in the zebrafish CMZ has several features in common with the neurogenic niches in the adult mammalian brain, including the subventricular zone (SVZ) and the dentate gyrus subgranular zone (SGZ), which have been described as 'displaced neuroepithelium'. Adult neural stem cells are specialized astrocytes that serve as both neural stem cells and niche cells; they extend processes to form apical junctions at the ventricular surface and also have extensive contacts with the basal lamina surrounding blood vessels [5,7]. The retinal stem cells in the zebrafish CMZ that co-express *rx1, pax6a *and *vsx2/Chx10 *contact both the apical surface and the basal lamina and are in close proximity to a large blood vessel (ora collection vessel) that is SSEA-1^+ ^(Fig. 7). Other characteristics that mimic neuroepithelial cells of the developing neural tube and retina include activation of the Notch signaling cascade (reflected by expression of *notch1a/1b, notch3, dlA, dlB, dlC, her6/Hes1, her2/Hes5*: RLB and PAR, unpublished observations) and diffuse distribution of N-cadherin on the plasma membrane [52]. The radial glial marker BLBP is a downstream target of Notch signaling [71] and of Pax6 [72], both of which are implicated in the maintenance of neural stem cell fate. The CMZ cells in zebrafish express the radial glial marker BLBP, as do the cells of the ciliary epithelium and retinal pigmented epithelium, and immature Müller glia. Mitotic activity in the CMZ is stimulated by insulin-like growth factor (IGF) in goldfish [64], and by Wnt or sonic hedgehog (Shh) in chick, mouse and Xenopus retinas [26,73,74]. The SSEA-1 carbohydrate binds Wnts [8,49], and its association with the ora collection vessel in zebrafish may contribute to the special microenvironment that maintains a neuroepithelium at the CMZ. The proximity of the RPE on the apical side is another potential source of secreted signals that might promote neuroepithelial properties in the CMZ. The RPE in zebrafish expresses BLBP and is a source of Shh [75]. We have not yet looked for PEDF (pigment epithelium-derived factor) in zebrafish, but this growth factor is secreted by components of the SVZ and supports self-renewal of adult neural stem cells in mice [76].

A separate type of retinal stem cell niche is created within the differentiated retina by some of the Müller glia. Retinal Müller glia are often compared with radial glia of the developing brain [77]. Like radial glia, processes of Müller glia span the width of the retinal epithelium from the apical epithelial surface (outer limiting membrane) to the basal lamina at the vitreal surface and they express structural and functional markers of astrocytes (Fig. 7). In the intact retina of teleost fish, rapidly proliferating INL progenitors (the transit amplifying cells in the rod photoreceptor lineage) are clustered around radial Müller cell fibers and they use these radial processes as guides along which to migrate into the ONL [36,64], as we showed previously by serial reconstruction electron microscopy [35]. Not all Müller glia are associated with INL progenitors, and the frequency is highest near the retinal periphery (the youngest retina, most recently generated by the CMZ) [36,64]. We do not yet know whether these neurogenic niches are associated with a specialized subpopulation of Müller glial cells. In the intact retina, the retinal progenitors in these putative Müller cell niches are directed exclusively into the rod lineage, but following injury, the frequency of neurogenic clusters increases [78], the fate of the retinal progenitors is altered [65], and the structural and molecular characteristics of the Müller glia change [41,79] (present data). Müller glia in the injured fish retina exhibit many phenotypic changes associated with reactive gliosis, such as up-regulation of GFAP [42,55], which is often considered an indicator of stress and pathological processes [80] and reentry into the cell cycle [58]. Here we show that they also exhibit phenotypic changes suggestive of dedifferentiation, such as re-expression of BLBP and migration of their nuclei toward the apical surface, both of which are characteristic of immature Müller glia in the larval zebrafish retina (RLB, LKB and PAR, unpublished observations). The injury-induced retinal stem cells/multipotent progenitors are entwined in Müller processes, which in response to injury up-regulate components of the Notch-Delta signaling pathways and *N-cadherin *(*cdh2*) [81]. The N-cadherin protein becomes distributed to the basolateral membranes of the activated Müller glia, similar to its distribution on neuroepithelial cells.

Radial glia in the embryonic mammalian brain are neurogenic progenitors [6,82,83] and specialized astrocytes in the adult mammalian brain function as neural stem cells [2,4,84], so it is not surprising that we and others have suggested that Müller glia might generate the injury-induced retinal stem cells in teleost fish [41,42,66,79], although direct evidence that employs a selective and permanent lineage tracer for Müller glial fate mapping has not yet been accomplished. Fischer and Reh [57,85] have proposed that a latent neurogenic capacity persists in Müller glia of early postnatal chick based on observations of BrdU incorporation and expression of neuronal markers in immature Müller cells following intraocular injection of neurotoxins (NMDA, kainate or colchicine) or growth factors (IGF and FGF2), and similar observations were made in rat retina [86]. However, physiologically relevant, regenerative repair of retinal neuron loss has only been demonstrated in teleost fish [40] and some amphibians [26].

One of the most striking morphological changes in the injured fish retina is the migration of proliferating retinal progenitor cells and Müller nuclei to the apical surface and the subsequent reestablishment of a neuroepithelium; this anatomical organization is a necessary condition for regeneration of retinal neurons independent of the mechanism of injury [39,55,63]. For example, neurotoxins that selectively destroy retinal neurons in the inner layers of adult goldfish fail to elicit a retinal stem cell response and the missing neurons are not replaced. Only when photoreceptors are destroyed is the regenerative process triggered and the retina repaired including inner neurons as well as photoreceptors. One possible explanation for this differential response to injury is that damaged photoreceptors release factors that stimulate retinal stem cells and create a 'regeneration niche'. Another possibility, not mutually exclusive, is that in the absence of photoreceptors, the proliferating retinal progenitors reestablish contact with the apical surface thus recreating a neuroepithelial organization similar to that found in the CMZ. Migration of Müller nuclei to the apical surface following loss of photoreceptors is clearly not a sufficient trigger to elicit a regenerative stem cell response, since Müller glia in the mammalian retina undergo a similar behavior in the injured or dystrophic mammalian retina but do not generate neurons [87]. The key unanswered question is what extrinsic signals are necessary and sufficient to provoke the formation of a regenerative retinal stem cell niche? Several extracellular factors associated with maintenance of the undifferentiated, multipotent state in neural progenitors in early development, promote glial differentiation at later stages, including activation of LIF and Notch signaling [88,89]. In the developing retina, activation of Notch signaling either maintains undifferentiated progenitors or promotes Müller glia differentiation, depending on stage-dependent contextual cues [45,46,90]. The LIF pathway may also be implicated: Müller glia isolated from the neonatal mouse retina and induced to proliferate by exposure to growth factors express LIF [91], and LIF is necessary for injury-induced neurogenesis in mouse olfactory epithelium [92].

## Conclusion

Our data indicate that Müller glia in the adult fish retina have a complex response to local injury that includes some features of reactive gliosis (up-regulation of GFAP and re-entry into the cell cycle) together with dedifferentiation and re-acquisition of phenotypic and molecular characteristics of multipotent retinal progenitors (diffuse distribution of N-cadherin, activation of Notch-Delta signaling, and expression of *rx1, vsx2/Chx10*, and *pax6a *and BLBP). The outcome of this injury-induced glial activation is not generation of more Müller glia and formation of a glial scar, as is typical in mammalian retina [80,87], but instead a retinal stem cell niche is recreated with molecular and morphological features that mirror the neurogenic CMZ. Our data suggest that in the injured retina activated Müller glia cells define the retinal stem cell niche and our results are consistent with the proposal that retinal stem cells derive from proliferating Müller glia. This idea fits with the emerging view that adult neural stem cells *in vivo *are a subpopulation of radial glial-derived cells that retain a neurogenic potential.

## Methods

### Thermal retinal lesions

Zebrafish from our outbred wild-type colony were maintained according to standard procedures. Adult fish were anesthetized in 0.04% Tricaine (3-amino benzoic acidethylester; Sigma-Aldrich, St. Louis, MO) and positioned on their sides on the stage of a stereomicroscope. The dorsal scleral surface of the eyeball was exposed by exerting pressure on the ventral eye to torque it in its socket. A 0.2–0.3 mm diameter copper wire was attached with an alligator clip to a soldering pen (model #64-2055A, Radio Shack, Fort Worth TX), which was mounted on a micromanipulator and set at 15-watts. The heated copper wire was touched to the outer surface of the sclera for 5 seconds. Fish were revived and allowed to recover for 1 to 31 days post-lesion (dpl). The University Committee on Use and Care of Animals at the University of Michigan approved all experimental procedures.

### Bromodeoxyuridine labeling of proliferating cells

Two methods were used to expose retinal cells to the thymidine analog, 5-bromo-2'-deoxyuridine (BrdU; Sigma-Aldrich). Zebrafish from 1 to 10 dpl were injected intraperitoneally with BrdU [66]. Alternatively, fish were placed for 2 hours in aquarium system water containing 5 mM BrdU [41]. From 4 hours to 31 days later, fish were killed and the eyes prepared for histology as described below.

### Histology and immunocytochemistry

For histology, lesioned eyes were enucleated, fixed in 2.5% glutaraldehyde/1%paraformaldehyde in 0.08 M phosphate buffer, 3% sucrose, 1 mM MgSO_4 _(pH 7.4) and embedded in Eponate 12 Resin (Ted Pella, Redding, CA). Semithin (1 μm) sections were stained with 1% methylene blue, 1% azure II in 1% sodium borate.

For immunocytochemistry, larval zebrafish were treated with 0.2 mM PTU (1-phenyl-2-thiourea) starting at ~10 hpf to block formation of melanin in the RPE. Intact larval fish and juvenile (2-month-old) fish and enucleated eyes from and adult fish (≥ 1-year-old) were fixed in 4% paraformaldehyde in 0.1 M phosphate buffer and prepared for cryosectioning as described previously [93]. Tissue used for PCNA (proliferating cell nuclear antigen) immunocytochemistry was fixed with 4% paraformaldehyde in 95% ethanol. Slides used for BrdU immunocytochemistry were pre-treated with 2N HCl for 30 min. Some slides were processed for antigen retrieval by incubation for 20 mins. at 98 C in 10 mM sodium citrate, pH 6.0, with 0.05% Tween20, then cooled and rinsed in 0.1 M phosphate buffered saline.

Primary antibodies and dilutions included: Monoclonal antibodies – anti-PCNA (Sigma-Aldrich), 1:1000; zrf1 (Zebrafish International Resource Center, ZIRC, Eugene, OR), 1:5; zpr1 (ZIRC), 1:200; rat anti-BrdU (Accurate Chemical, Westbury, NY), 1:50; 4C4 (from Jonathan Scholes, University College, London), 1:200; SSEA-1/LeX, carbohydrate epitope (MC-480, Developmental Studies Hybridoma Bank, Iowa City, IA), 1:5. Polyclonal antibodies – anti-human GFAP (DakoCytomation, Carpinteria, CA), 1:100; anti-human BLBP (Abcam, Cambridge, MA), 1:1000; affinity purified, anti-zebrafish N-cadherin (N-terminal peptide) [52], 1:500; anti-GFP (Invitrogen, Carlsbad, CA), 1:200.

Secondary antibodies used at 1:200 dilution included: anti-mouse IgG, Alexa 488 conjugate; anti-rat IgG, Alexa 647 conjugate; anti-rabbit IgG, Alexa 555 conjugate (Alexa conjugates from Invitrogen); anti-mouse IgM, FITC (fluorescein isothiocyanate)-conjugate; anti-mouse IgG, Cy-3, FITC and AMCA (7-amino-4-methylcoumarin-3-acetic acid) conjugates (Jackson ImmunoResearch Laboratories, Inc., West Grove, PA). Slides were mounted in ProlongGold antifade reagent (Invitrogen), premixed with the nuclear stain, DAPI (4', 6-diamidino-2-phenylindole).

### In situ hybridization

Methods for synthesis and hybridization of digoxigenin (DIG)-labeled or fluorescein (FL)-labeled RNA probes [94] were modified as follows: Hauptmann hybridization buffer [95], hybridization temperature at 65 C, no RNase treatment, and increased posthybridization stringency [96]. Probes transcribed from zebrafish cDNA templates (minimum length: 741 bp) were used at the concentrations indicated: *notch1a*, *notch1b*, *notch2*, *notch3 *(from Michael Lardelli, University of Adelaide) 0.8 μg/ml; *her1, her6 *(from Jose Campos-Ortega, deceased) 8 μg/ml; *deltaA*, *deltaD *(from Bruce Appel, Vanderbilt University) 8 μg/ml; *deltaB*, *deltaC *(from Julian Lewis, University College London) 8 μg/ml; *rx1, rx2, rx3 *(from Peter Mathers, University of West Virginia) 4 μg/ml; *vsx2 *(from Nisson Schechter, SUNY Stony Brook) 4 μg/ml; *pax6a *(from Nadean Brown, Cincinnati Children's Hospital) 1 μg/ml; *gfap *(from James Warren, Penn State) 8 μg/ml; *ascl1a-DIG *(from Nadean Brown) 4 μg/ml; *neuroD *(from Peter Hitchcock, University of Michigan) 4 μg/ml; *apoE *(from Susan Lyons, University of Michigan) 0.5 μg/ml; cone opsin genes *sws1, sws2, rh2-1, lws-1 *(from Thomas Vihtelic, University of Notre Dame), 250 ng/ml.

For two-color *in situ *hybridization [94] DIG-labeled and FL-labeled probes were mixed and hybridized to the tissue, then detected with anti-DIG or anti-FL antibodies conjugated to alkaline phosphatase (AP) or horseradish peroxidase (POD), respectively. Fast Red (FR) color substrate for AP (Roche Applied Science, Indianapolis, IN) was followed by the indirect Tyramide Signal Amplification (TSA) fluorescence system (TSA-biotin/avidin-FITC) to detect the POD-conjugated antibody (Perkin Elmer Life and Analytical Sciences, Boston, MA). Alternatively, to avoid any possibility that the dense FR precipitate would obscure the TSA-FITC signal, both anti-FL and anti-DIG antibodies were conjugated with POD. The FL-labeled probes were detected first by incubation with the anti-FL POD conjugated antibody followed by indirect TSA-biotin/avidin-FITC, as above. The POD was then inactivated by incubation with 3% H_2_O_2 _in 0.1 M PBS for 15 minutes, and sections were incubated with the anti-DIG POD-conjugated antibody and detected using direct TSA-Cy3 (Perkin Elmer).

### Cell counts

Fish with lesioned retinas were injected intraperitoneally with BrdU at 3, 5, 7 or 10 dpl, retinas were fixed at 31 dpl and cryosectioned. DIG-labeled probes for zebrafish cone opsin genes (*sws1, sws2, rh2-1, lws-1*) were combined at a concentration of 250 ng/ml each, hybridized, and detected with anti-DIG (AP-conjugate visualized with FR), followed by BrdU immunofluorescent detection.

To determine the time course of photoreceptor regeneration, the area with regenerated photoreceptors was identified by BrdU^+ ^nuclei in the INL. Labeling by *in situ *hybridization with the cone opsin cocktail identified cone photoreceptors, and those with BrdU^+ ^nuclei were counted. BrdU^+ ^rod nuclei within the segment of retina delimited by BrdU label in the INL were also counted. (Note that BrdU^+ ^rod photoreceptors outside the lesioned zone are not regenerated neurons but instead are newly added in conjunction with normal retinal growth in teleost fish. These were not counted.) Between 4 and 17 sections per lesion were scored and the mean number of BrdU^+ ^cones and rods per section was calculated.

To quantify the time course of proliferation in injury-activated Müller glial cells, retinas from fish labeled with a 2-hour pulse of BrdU at 2, 3 or 4 days post-lesion were processed for *in situ *hybridization with the Müller marker *apoE*. BrdU^+ ^nuclei were counted in 2 to 9 sections from each retina and scored for double-labeling with ApoE. To maximize the number of BrdU^+ ^nuclei in each sample we examined sections cut tangentially through the lesion.

### Imaging

For light microscopy we used epifluorescent compound microscopes (Zeiss Axioplan equipped with an Axiophot 2 camera or Zeiss AxioImager and ApoTome with an AxioCam MRM camera) supported by AxioVision software (Carl Zeiss, Jena, Germany), or an Olympus BX-51 epifluorescent compound microscope (Olympus, Tokyo, Japan). For laser scanning confocal microscopy we used an Olympus Fluoview 500, equipped with 405 nm blue diode, 458 nm, 488 nm, and 514 nm multiline argon, 543 nm helium neon green and 633 nm helium neon red lasers. Images were processed with Fluoview version 4.3 with Tiempo software. We used Adobe PhotoShop to adjust digital images for brightness, contrast and sharpness. Hues in RGB color space were adjusted with the Channel Mixer tool. Overlays of multiple fluorescent and differential interference contrast images from the same field of view were generated in PhotoShop with the Layer Style tool by selecting the Screen or Lighten option for the Blend Mode.

## Authors' contributions

PAR conceived of and designed the study, wrote the paper and prepared the figures. LKB invented the heat lesion method, processed and photographed most of the histological preparations for immunocytochemical and in situ hybridization, and carried out and analyzed the cell counts. RLB processed and photographed some of the *in situ *hybridization preparations and created the Tg(*gfap*: GFP) transgenic zebrafish. JJP produced and analyzed the regional gene expression data in Table 1. All authors have read and approved the final manuscript.
